# *ChPDIA3* targeted by miR-126-x and miR-21-y responds to *Vibrio harveyi* infection in *Crassostrea hongkongensis*

**DOI:** 10.3389/fcimb.2025.1533154

**Published:** 2025-02-14

**Authors:** Yongkang Hou, Fangqi Zhang, Xiaokun Liu, Dongming Huang, Zhimin Li

**Affiliations:** College of Fishery, Guangdong Ocean University, Zhanjiang, Guangdong, China

**Keywords:** *Crassostrea hongkongensis*, *Vibrio harveyi*, PDIA3, miRNA regulation, immune response

## Abstract

**Introduction:**

The Hong Kong oyster (*Crassostrea hongkongensis*), as the main marine aquaculture shellfish in the South China Sea, not only has high economic and ecological value, but also is an ideal model for conducting research on pathogen-host interactions. In the cultivation process of *C. hongkongensis*, there is a challenge posed by *vibrios*. To improve the antibacterial strains of *C. hongkongensis*, we have studied the gene associated with immunity, *PDIA3*.

**Methods and findings:**

In this study, we cloned the *PDIA3* sequence of the *C. hongkongensis*, using the RACE technique. It has a total of 2081 bp and contains a 5'-UTR of 55 bp and a 3'-UTR of 547 bp. The ChPDIA3 gene sequence has an ORF frame that is 1479 bp in length and encodes 492 amino acids. Analysis of the phylogenetic tree constructed by Neighbor Joining method showed that *ChPDIA3* clustered with other shellfishes into a single unit, which was consistent with the law of species evolution.

**Discussion:**

The highest expression of *ChPDIA3* was detected in gill tissues of the *C. hongkongensis* using RT-qPCR, and significantly higher expression in *V. harveyi* and LPS infection than Poly(I:C) (*P*<0.05). This may indicate that *ChPDIA3* is primarily involved in the immune response against bacterial infections in the *C. hongkongensis*. The binding sites of miR-126-x, miR-21-y and *ChPDIA3* were detected using dual luciferase experiments, respectively. The results showed that both miR-126-x and miR-21-y inhibited the 3'-UTR region of *ChPDIA3*. This suggested that both miR-126-x and miR-21-y inhibited *ChPDIA3* expression. This study will help to further understand the function of *ChPDIA3* in response to pathogen infection, thus providing new ideas for understanding the resistance and adaptation of the *C. hongkongensis* to *Vibrio* infection.

## 1 Introduction

The *Crassostrea hongkongensis* is mainly distributed in the coastal areas of the South China Sea, including the coastal areas of South China and Vietnam (Peng et al., 2021). It is worth noting that *C. hongkongensis* have been cultivated in the coastal areas of southern China for nearly a thousand years. The *C. hongkongensis* is a typical intertidal species whose living environment switches between being exposed to air and being inundated by seawater on a daily basis, thus it has a strong ability to adapt to the environment (Zhang et al., 2012). In addition, *C. hongkongensis* not only has a positive impact on the ecosystem, but also has high commercial value. For example, it is a high protein, low-fat aquatic product, rich in various vitamins, glycogen, fatty acids, essential amino acids, and micronutrients such as calcium, zinc, copper, selenium, etc., so it is highly popular in the market (Grabowski et al., 2012; Loaiza, 2023). However, the rapid development of the aquaculture industry has been accompanied by a gradual deterioration of the aquaculture environment, and shellfish aquaculture has been challenged by pathogenic microorganisms such as bacteria and viruses (Fehrenbach et al., 2024; Li et al., 2023; Shingai, 2024). Due to environmental influences, violent deaths occur frequently, causing huge economic losses to the entire farming industry. The sustained development of oyster farming has brought pressure to the marine environment, especially the spread of *Vibrio* disease in oysters has had a negative impact on the development of oyster farming (Dégremont, 2021; Oyanedel, 2023; Yu et al., 2023).

*Vibrio harveyi*, a Gram-negative bacterium, was first isolated from *Carcharhinus plumbeus* and *Negaprion brevirostris*, which was very widely distributed in the oceans (Colwell and Grimes, 1984; Grimes et al., 1984; Muthukrishnan et al., 2019). *V. harveyi* is a common conditional pathogen in aquaculture and has been widely recognized as a common pathogen of many commercially farmed fish (Angthong et al., 2023; Dong et al., 2017; Firmino et al., 2019; Wang et al., 2022), mollusks (Liu et al., 2014; Morot et al., 2021) and crustaceans (De Souza Valente and Wan, 2021; Rungrassamee et al., 2016) in warm waters. Some studies have shown that *V. harveyi* spreads mostly during the hot summer months, mainly attacking damaged body surfaces or the digestive tract of animals. *V. harveyi* infection causes ulcers, tissue necrosis and massive epithelial cell death in animals (Yuan et al., 2023). In addition, it triggers the generation of oxidative stress, apoptosis, and inflammatory responses in the body (Angthong et al., 2023; Chen et al., 2023; Hao et al., 2023). In summary, *V. harveyi* kills large numbers of marine vertebrates and invertebrates (Austin and Zhang, 2006; Travers et al., 2008).

Protein Disulfide Isomerase Family A Member 3 (PDIA3), also known as ERp57, is one of the members of the PDI family and is located mainly in the endoplasmic reticulum (Luo et al., 2008). PDIA3 is thought to be a protein disulfide isomerase involved in the proper folding and processing of proteins within the endoplasmic reticulum. It can maintain protein structure and function by catalyzing the formation and rearrangement of disulfide bonds (Hetz et al., 2006; Yoneda et al., 2001). *PDIA3* may be involved in intracellular redox homeostasis. It can interact with other oxidoreductases to regulate intracellular oxidative stress and protect cells from oxidative damage (Chamberlain et al., 2019). Alternatively, *PDIA3* may play a role in endoplasmic reticulum stress response, helping cells to cope with endoplasmic reticulum stress and maintain their homeostasis (Mahmood et al., 2021). Abnormal expression or function of *PDIA3* has been found to be associated with certain diseases, such as inflammatory response (Wang, 2019), neurodegenerative diseases (Zhu et al., 2024), and cancer (Wang et al., 2023). In aquatic animals, the *PDIA1* and *PDIA3* of *Dicentrarchus labrax*, *L*. have been cloned entirety (Pinto et al., 2013). Moreover, *ERp57* of Oncorhynchus mykiss was cloned and expressed in various tissues (Sever et al., 2013). Furthermore, it has been shown that *PDIA3* is differentially expressed in *O. mykiss* in the presence of *Vibrio anguillarum* infection (Yang, 2023). In our previous study, we sequenced the transcriptome and miRNAome of the *C. hongkongensis* under *V. harveyi* infection, and the results showed that miR-126-x with miR-21-y had a regulatory effect on *PDIA3* (Hou et al., 2023, 2024). To verify the regulatory relationship between miR-126-x and miR-21-y on *PDIA3* in more depth, in this study, it is proposed to clone the full length of *PDIA3* from the *C. hongkongensis*, using RACE technology, and their sequences were analyzed by bioinformatics. The expression pattern of *PDIA3* was analyzed using RT-qPCR technology and RNA interference. Validation of miR-126-x and miR-21-y as having regulatory effects on *PDIA3* using a dual luciferase reporter. This will provide new ideas for understanding the improvement of the resistance and adaptability of the *C. hongkongensis* to *Vibrio* infections, as well as for realizing the scientific culture and industrial development of the *C. hongkongensis*.

## 2 Materials and methods

### 2.1 Collection of *C. hongkongensis* samples

The *C. hongkongensis* used in the experiment were all of 2 years old, purchased from the seafood market in Xiashan District, Zhanjiang City, and temporarily reared for one week in the Shellfish Genetics and Enrichment Laboratory of Guangdong Ocean University. *Chlorella* was fed during the temporary incubation period, the water temperature was 28°C, the salinity was 20 ppt, continuous aeration was maintained. 108 C*. hongkongensis* were randomly selected, with a mean weight of 201.65 ± 22.20 g and a mean shell height of 117.03 ± 11.16 mm. They were divided into 4 groups, i.e., PBS group, *Vibrio* group, LPS group, and Poly(I:C) group, and each group was set up in 3 parallels, with 15 C*. hongkongensis* placed in each parallel group. *C. hongkongensis* in the *Vibrio* group were injected with 200 μL of *V. harveyi* solution at a concentration of 1×10^8^ CFU·mL^-1^. The LPS group was injected with 200 μL of LPS (Solarbio, purity≥98%) solution at a concentration of 10 μg·mL^-1^. The Poly(I:C) group was injected with 200 μL of Poly(I:C) solution at a concentration of 5 mg·mL^-1^. The control group was injected with an equal amount of PBS. At 0, 12, 24, 48 and 72 h after injection, 2 C*. hongkongensis* were randomly selected from each parallel group and their hemolymph, gill, mantle, digestive diverticulum, adductor muscle, and gonad were sampled. All tissue samples were collected under strict sterile conditions. After collection, the samples were rapidly immersed in liquid nitrogen for snap-frozen to preserve their biological activity. They were then transferred to a -80°C low-temperature environment for storage in preparation for subsequent experiments.

### 2.2 Total RNA extraction and cDNA synthesis

At each time point within each group, three *C. hongkongensis* individuals were randomly selected, and RNA was extracted from the hemolymph, gill, mantle, digestive diverticulum, adductor muscle, and gonad. The extraction was performed using the TransZol Up Plus RNA Kit (TransGen Biotech), following the manufacturer’s instructions. RNA concentration and purity were assessed using a NanoDrop 2000 spectrophotometer. RNA integrity was determined by agarose gel electrophoresis. All RNA samples were reverse transcribed using the EasyScript^®^ One-Step gDNA Removal and cDNA Synthesis SuperMix kit (TransGen Biotech), following the instructions.

### 2.3 Cloning of *ChPDIA3*

We screened the intermediate sequence of the *ChPDIA3* gene based on the available transcriptome data (GenBank project accession PRJNA999463), and then used this sequence to design 5’RACE and 3’RACE specific primers for this gene using Primer Premier 5 (**Table 1**). Full-length amplification was performed using the SMARTer^®^ RACE 5’/3’ Kit Protocol-At-A-Glance (TaKaRa), following the instructions. PCR products were detected by agarose gel electrophoresis, then cut and recovered using the NucleoSpin^®^ Gel and PCR Clean-Up kit (TaKaRa), and the recovered products were ligated into pEASY-Blunt (TransGen Biotech) vector. Trans1-T1 competent cell (TransGen Biotech) were used for transformation, and the products were placed in solid medium and incubated for 12 h in a 37°C incubator. Single colonies were screened and placed in liquid culture at 37°C, 200 rpm for 12 h. Positive cloning was performed, and the positive cloning products matching the target bands were sent to Sangon Biotech Ltd. for sequencing.

**Table 1:** Primer information.

Primer name	Sequence	Application
ChPDIA3-3’	CAGCCAGAGACGAGAGAGACCGCA	RACE
ChPDIA3-5’	CATCATTGGCTGTGGCGTCCATTT	RACE
ChPDIA3-Sense	TGGAATGGGGGATGTCAGTGGA	RNA i
ChPDIA3-Anti Sense	CCCTTAGGAGCAAAATAGATTGTAGGGA	RNA i
ChPDIA3-F	AAAATGGACGCCACAGCC	RT-qPCR
ChPDIA3-R	CACGATCAAAGCCCGACAG	RT-qPCR
CALR-F	CTTTGGCAGGTGAAATCGG	RT-qPCR
CALR-R	CTCTTCTTTCTTTCTTCCTCATCCT	RT-qPCR
LMAN1-F	CATCCGATTAGCCCCGTCT	RT-qPCR
LMAN1-R	ACTGGTCCCTCCTGTCCCTT	RT-qPCR
LMAN2-F	ACCTACAGTAACCACAATGGACCT	RT-qPCR
LMAN2-R	TTTCGGAACTTAGCCTCGC	RT-qPCR
β-actin-F	CTGTGCTACGTTGCCCTGGACTT	RT-qPCR
β-actin-R	TGGGCACCTGAATCGCTCGTT	RT-qPCR
ChPDIA3-DLR-F	TGTTTAAACGAGCTCGCTAGCGTGAGGGGAGCAAGTTGTATAATTG	Vector construction
ChPDIA3-DLR-R	TGCCTGCAGGTCGACTCTAGAAAAAAAAAAAATAATTAAATTTATTTCCCA	Vector construction
miR-126-x mimics	CAUUAUUACUUUUGGUACGCG CGUACCAAAAGUAAUAAUGUU	Dual luciferase experiment
miR-21-y mimcis	CAACACCAGUCGAUGGGCUGU AGCCCAUCGACUGGUGUUGUU	Dual luciferase experiment
N.C mimics	UUGUACUACACAAAAGUACUGGUACUUUUGUGUAGUACAAUU	Dual luciferase experiment

### 2.4 Bioinformatics analysis

We used ORFfinder (https://www.ncbi.nlm.nih.gov/orffinder/) to identify open reading frame as well as sequence translations. The physicochemical properties of the amino acids were queried by the online software ProtParam (https://web.expasy.org/protparam/). The conserved domains were analyzed according to the protein information resource InterPro (https://www.ebi.ac.uk/interpro). The full-length protein sequences of the *PDIA3* homologues were downloaded at NCBI, and multiple sequence comparisons were performed using DNAMAN 5.0 with default parameters. Phylogenetic tree was constructed using Neighbor Joining in MEGA (v11.0.13).

### 2.5 Detection of *ChPDIA3* expression by RT-qPCR

Based on the sequences obtained from RACE cloning, primers were designed for RT-qPCR using Premier 5.0 (**Table 1**). Reactions were performed in Light Cycler 96, as described in the instructions for the PerfectStart^®^ Green qPCR SuperMix kit (TransGen Biotech). Each sample was processed in triplicate in Light Cycler 96. The 2^-ΔΔCt^ method was used to calculate the relative expression of the *ChPDIA3* gene in tissues of the *C. hongkongensis* such as hemolymph, gill, mantle, digestive diverticulum, adductor muscle, and gonad, with β-actin as the reference gene. The cDNAs of *Vibrio* group, LPS group, Poly(I:C) group and PBS group were taken as templates, and the rest of the conditions were the same as those mentioned above, to verify the expression of *ChPDIA3* at different time points in each group.

### 2.6 dsRNA synthesis

Based on the sequences obtained by RACE cloning, primers were designed using Premier 5.0 (**Table 1**) and PCR amplification was performed using Seq Amp DNA Polymerase (TaKaRa). Cutting gel recovery, ligation, transformation, positive cloning, and bacteriophage sequencing were performed as in Section 2.3. After the bacterial fluids were sequenced, a pair of forward and reverse sequences were selected for amplification. Plasmids obtained from amplification were extracted using the GeneJET Plasmid Miniprep Kit (Thermo). The plasmids were digested using Past I-HF enzyme and the digested products were detected by agarose gel electrophoresis. Products from single bands were purified using the GeneJET PCR Purification Kit (Thermo). The purified products were transcribed *in vitro* using the T7 RNAi Transcription Kit (Vazyme Biotech) as described in its instructions. The products of *in vitro* transcription were extracted by phenol-chloroform-ethanol absolute, then precipitated by ethanol absolute and finally dissolved in DEPC water.

### 2.7 RNA interference experiments and sample collection

The *C. hongkongensis* used in the experiment were temporarily reared for one week*. Chlorella* was fed during the temporary incubation period, the water temperature was 28°C, the salinity was 20 ppt, continuous aeration was maintained. 90 C*. hongkongensis* of similar growth condition and free from disease and injury were randomly selected. They were divided into three groups, i.e., RNA interference group, *Vibrio* group and control group, and three parallels were set in each group. From the pre-experiment, dsRNA acted 24 h after injection, so the experimental group was first injected with 100 μL of dsRNA (at a concentration of 1 μg·μL^-1^). After 24 h, 100 μL of *V. harveyi* was injected. The *Vibrio* group was injected with an equal amount of *V. harveyi* solution at a concentration of 1×10^8^ CFU·mL^-1^, and the control group was injected with an equal amount of 5 × PBS. 2 C*. hongkongensis* were randomly selected from each parallel group for sampling 12, 24 48 and 72 h after the completion of injection, respectively. The samples were first snap-frozen in liquid nitrogen and later stored at -80°C for later RNA extraction.

### 2.8 *ChPDIA3* expression pattern after RNA interference

At each time point in each group, three samples were randomly selected for RNA extraction and reverse transcription, which was performed in the same way as in Section 2.2. The cDNA at each time point was used as a template for RT-qPCR to detect the expression of *ChPDIA3* in *V. harveyi* infection after RNA interference.

### 2.9 Dual luciferase experiments

The relationship between *ChPDIA3* and miR-126-x and miR-21-y binding sites were selected for vector construction, respectively. The vector was prepared by Wuhan Zhibo Biotechnology Co., Ltd. and its sequence accuracy was verified. HEK293T cells were inoculated in 96-well plates at 2×10^4^ cells/well, with 3 replicate wells per group. The medium was 100 μl of DMEM-High Sugar medium containing 10% FBS, which was incubated overnight in a 5% CO_2_, 37°C incubator. The transfection system was configured according to **Table 2**. Solution A and solution B were mixed and left to stand for 20 min at common temperature. 20 μL of transfection complex was added to each well and then placed in 5% CO_2_, 37°C incubator. After 48 h of transfection, the old medium was aspirated and 100 μL of PLB (Passive Lysis Buffer) was added to each well of cells. Lysis was performed on a shaker at common temperature for 15 min. After adding 20 μL of cell lysate to the luminescent plate, background values were read for 2 s using Promega’s GloMax^®^-Multi multifunctional enzyme marker. Add 20 μL of LAR II working solution to each well, mix quickly, and read values for 2 s. After the reading is complete, add another 20 μL of Stop & Glo^®^ Reagent to each sample and mix quickly. Place in a luminescence detector, run the program and read the fluorescence value for 2 s. After completing the reading, save the data.

**Table 2:** Transfection systems for dual luciferase experiments.

Clusters		Dosage
A solution	Reporter plasmid	50 ng
	Mimics (20 μM)	0.5 μl
	Opti-MEM	10 μl
B solution	Opti-MEM	10 μl
	Lipofectamine2000	1 μl

### 2.10 Statistical analysis

All data analyses were performed using Origin 2024 and IBM SPSS Statistics 26.0. Samples from different groups at the same time were analyzed for statistical significance using Duncan’s Multiple Range Test, the significance level was set at 0.05.

## 3 Results

### 3.1 Bioinformatics of *ChPDIA3*

**Figure 1:**
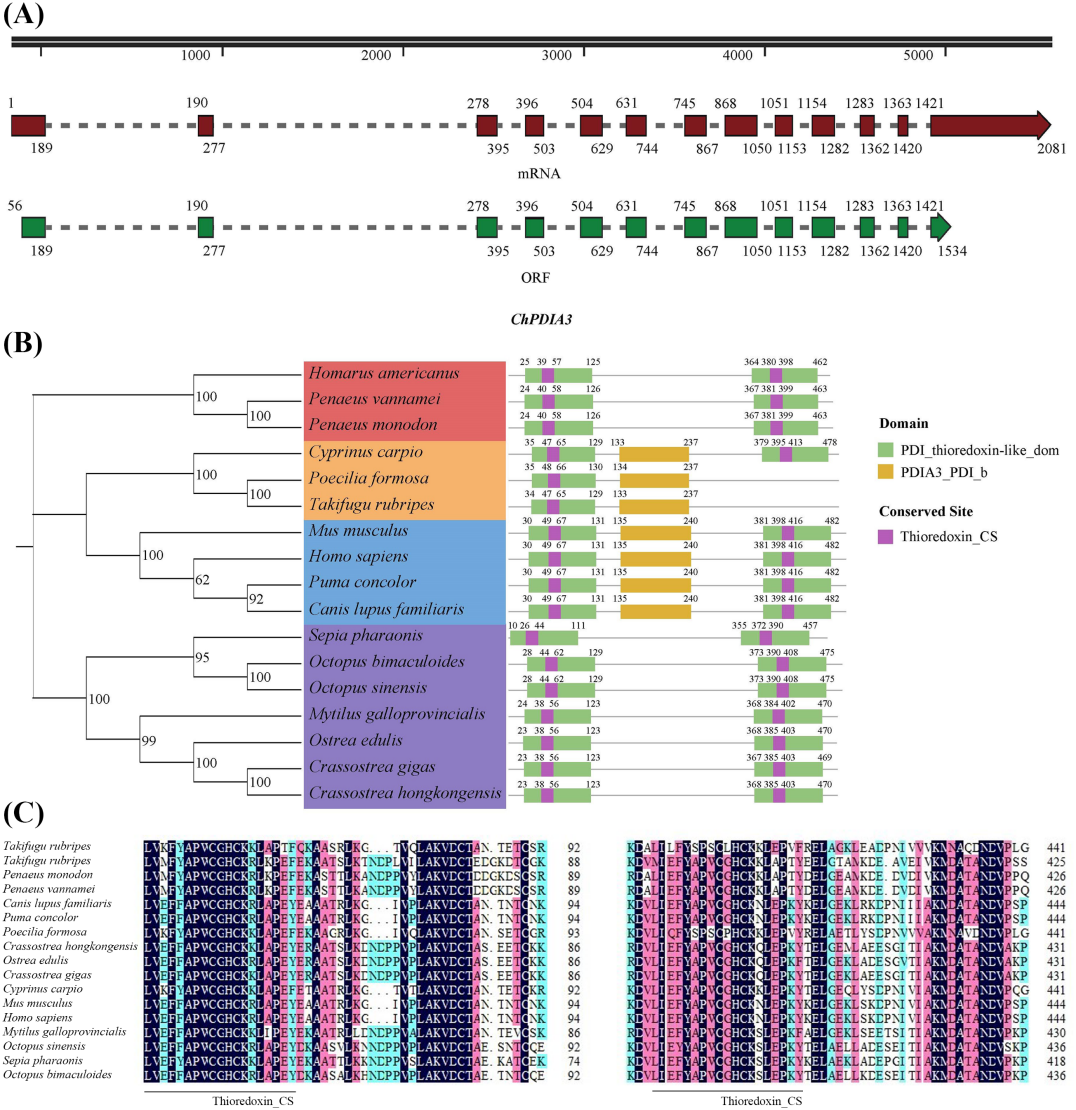
A *PDIA3* gene in *C*. *hongkongensis* and phylogenetic tree of the *ChPDIA3* protein. **(A)** Schematic presentation of *ChPDIA3*. Black line, structure of the gene in the genome. Dark red and dark green boxes, *ChPDIA3* transcript and its coding region. **(B)** Phylogenetic tree and structural information of selected *PDIA3* protein. GenBank accession number (NCBI): *Homarus americanus* (XP_042223300.1), *Penaeus vannamei* (XP_027212336.1), *Penaeus monodon* (XP_037788996.1), *Cyprinus carpio* (XP_018979324.2), *Poecilia formosa* (XP_007566541.1), *Takifugu rubripes* (XP_029700442.1), *Mus musculus* (NP_031978.2), *Homo sapiens* (NP_005304.3), *Puma concolor* (XP_025774328.1), *Canis lupus familiaris* (XP_535453.3), *Sepia pharaonic*(CAE1328800.1), *Octopus bimaculoides* (XP_014785522.1), *Octopus sinensis* (XP_029643538.1), *Mytilus galloprovincialis* (VDI57500.1), *Ostrea edulis* (XP_048735080.2), *Crassostrea gigas* (XP_011453191.2). **(C)** Alignment of partial *PDIA3* domain containing active conserved site Thioredoxin_CS.

The cDNA sequence of the *PDIA3* gene of the *C. hongkongensis*, named *ChPDIA3*, has been cloned using the RACE technique. *ChPDIA3* has a total of 2081 bp, containing 55 bp of 5’-untranslated region (UTR) and 547 bp of 3’-UTR. The sequence of *ChPDIA3* has an ORF with a length of 1,479 bp, encoding 492 amino acids (**Figure 1A**). The molecular weight is 55530.92 Da, the theoretical pI is 5.48, and the grand average of hydropathicity is -0.551<0, which makes it a hydrophilic protein. The results of the phylogenetic tree showed that the *C. hongkongensis* was clustered with other shellfish and was genetically distant from crustaceans and vertebrates. The predicted conserved structural domains of the *ChPDIA3* sequence showed that the PDI_thioredoxin-like_dom are located at 23-123 aa and 368-470 aa (**Figure 1B**). Thioredoxin_CS in the *ChPDIA3* protein has the closest homology to the *C.gigas* and the Ostrea edulis compared to homologs from other species (**Figure 1C**).The cDNA sequence of *ChPDIA3* has been submitted to NCBI (GenBank: PP530092).

### 3.2 Expression of *ChPDIA3* in different tissues

**Figure 2:**
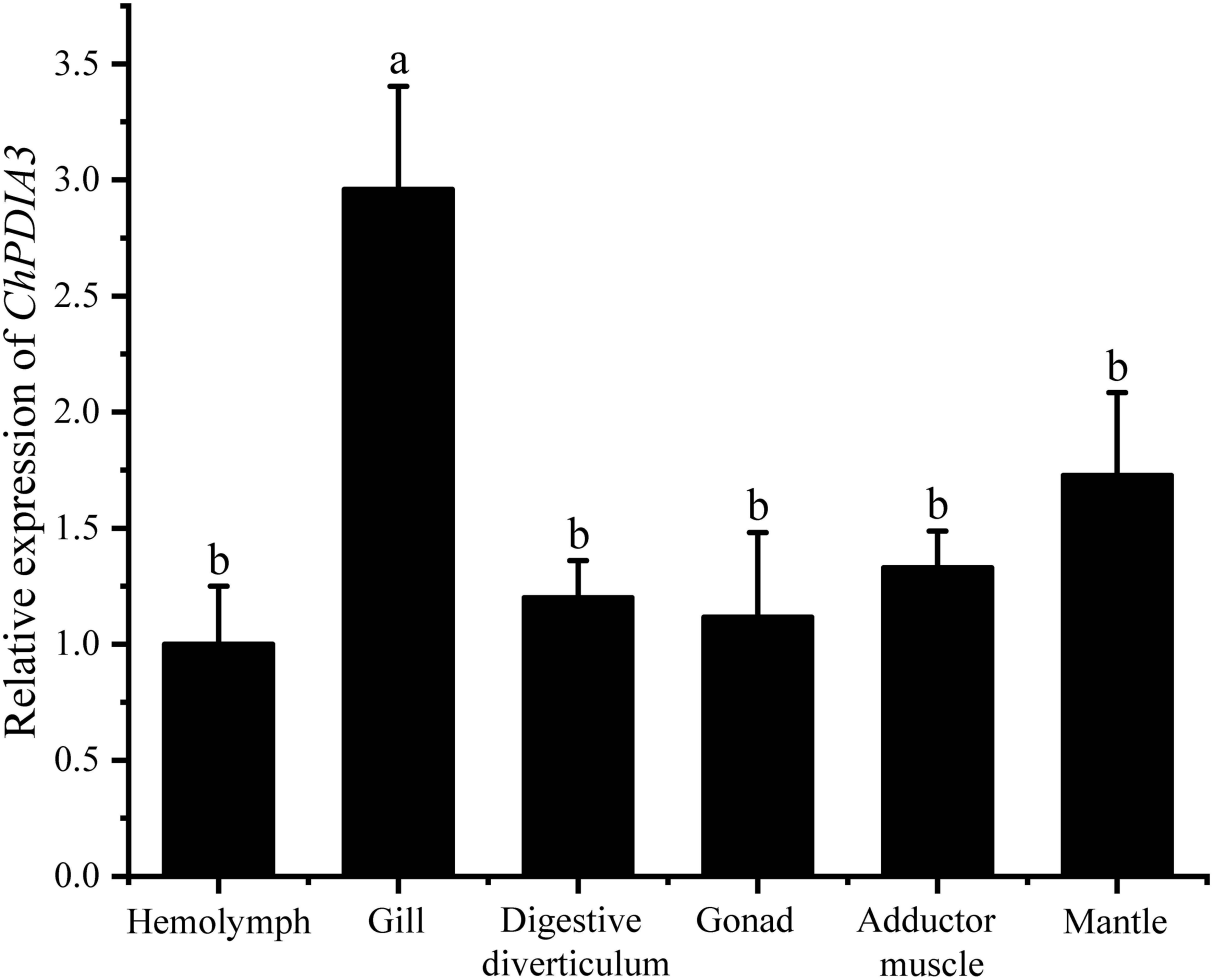
Expression of *ChPDIA3* in different tissues of the *C. hongkongensis*. Different lowercase letters indicate significant differences between treatment groups (*P*<0.05).

The expression of *ChPDIA3* in different tissues was examined using RT-qPCR, and the results are shown in **Figure 2**. *ChPDIA3* was expressed in tissues such as hemolymph, gill, mantle, digestive diverticulum, adductor muscle and gonad. And the highest expression was found in gill (*P*<0.05). Therefore, gills were targeted for subsequent studies.

### 3.3 Expression pattern of *ChPDIA3* in gill

**Figure 3:**
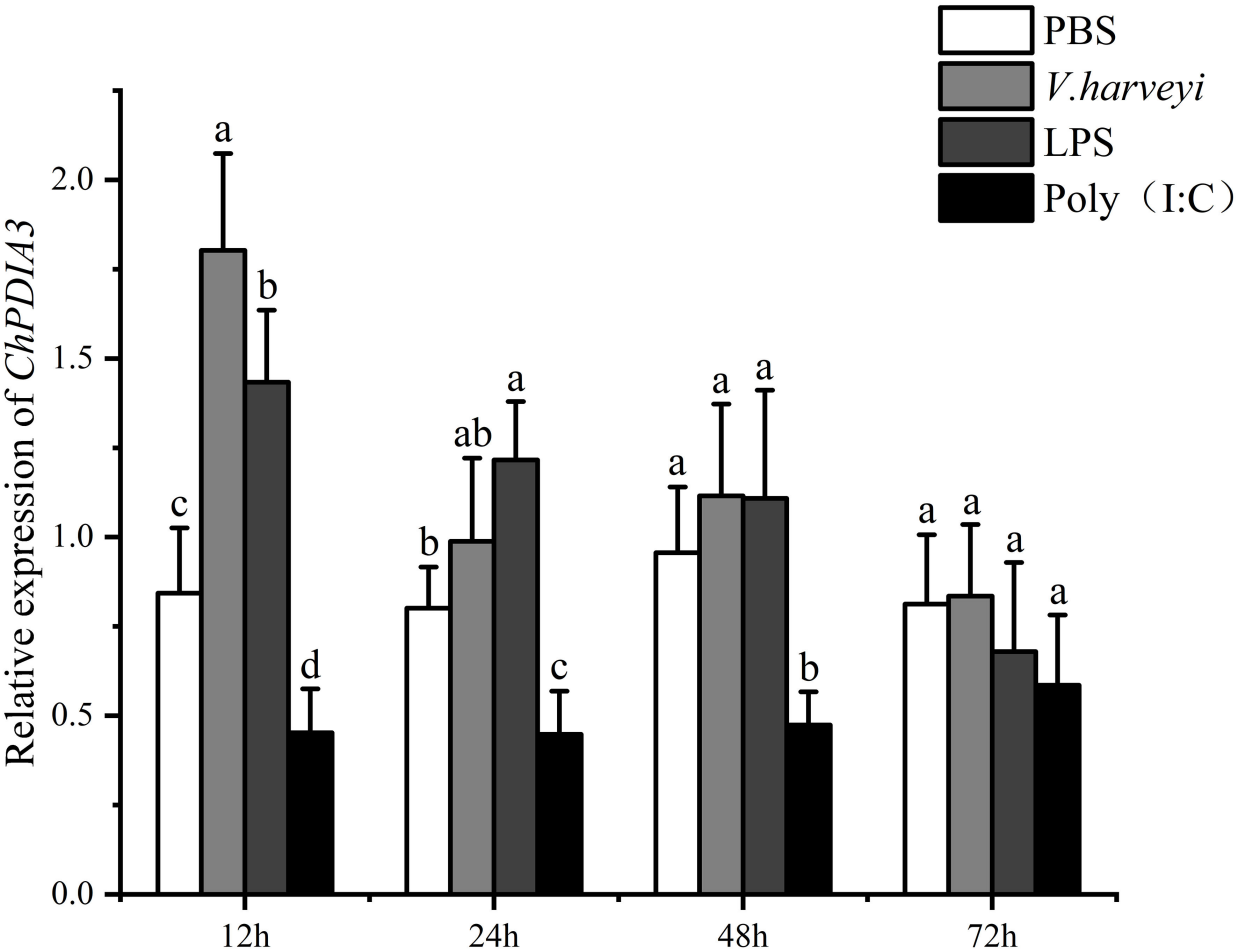
Expression pattern of *ChPDIA3* in different treatment groups. Different lowercase letters indicate significant differences between treatment groups (*P*<0.05).

The relative expression of *ChPDIA3* in the gill tissues of the *C. hongkongensis* under PBS, *V. harveyi*, LPS and Poly (I:C) treatments, at different time points, was examined using RT-qPCR. The results are shown in **Figure 3**, under *V. harveyi* and LPS treatments, the expression of *ChPDIA3* was significantly higher that of the other groups (*P*<0.05). And the highest expression at 12h after treatment, after which the expression of *ChPDIA3* gradually decreased. The expression of *ChPDIA3* in the gills of *C. hongkongensis* treated with Poly(I:C) was significantly lower than that of the PBS group (*P*<0.05), but showed a gradually increasing trend.

### 3.4 *ChPDIA3* expression in *V. harveyi* infection after RNA interference

**Figure 4:**
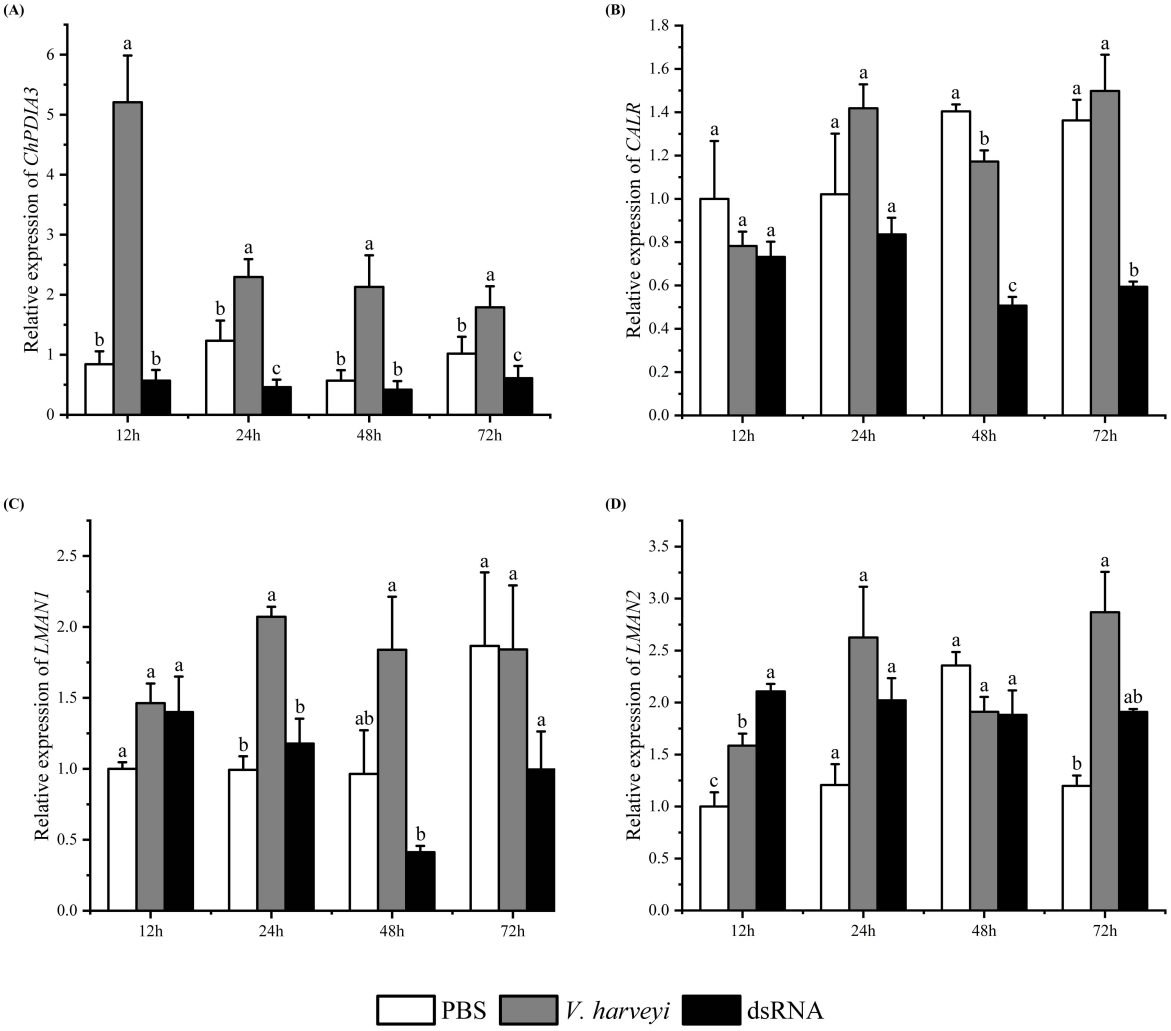
Expression patterns of genes after RNA interference. **(A)** shows the expression of the ChPDIA3 gene at different time points (12h, 24h, 48h, 72h) in the RNA interference group, the Vibrio group (injected with Vibrio harveyi), and the control group (injected with PBS) after RNA interference. **(B–D)** display the expression trends of downstream genes of ChPDIA3 (such as CALR, LMAN1, and LMAN2) in the endoplasmic reticulum protein processing pathway at different time points when ChPDIA3 is silenced by dsRNA. Different lowercase letters indicate significant differences between treatment groups (P<0.05).

The expression of *ChPDIA3* after silencing by dsRNA was detected by RT-qPCR. The results showed that the expression of *ChPDIA3* was significantly lower than that of *C. hongkongensis* injected with PBS and *V. harveyi* at all four time points (*P*<0.05). The inhibitory effect was most significant at 12 h of *V. harveyi* injection (*P*<0.05), and the inhibitory effect gradually failed after 72 h of injection, when the expression started to increase (**Figure 4A**). In the protein processing in endoplasmic reticulum pathway, the expression of its downstream genes was also downregulated when *ChPDIA3* was silenced by dsRNA. Except for *LMAN2*, the expression levels of the other two genes did not show significant differences at 12 hours (*P>*0.05). It was not until 48 hours that the expression level of *CALR* exhibited significant differences (*P*<0.05). Furthermore, the expression levels of *LMAN1* and *LMAN2* became progressively less significant compared to the control group (*P>*0.05) (**Figures 4B–D**).

### 3.5 Dual luciferase report

**Figure 5:**
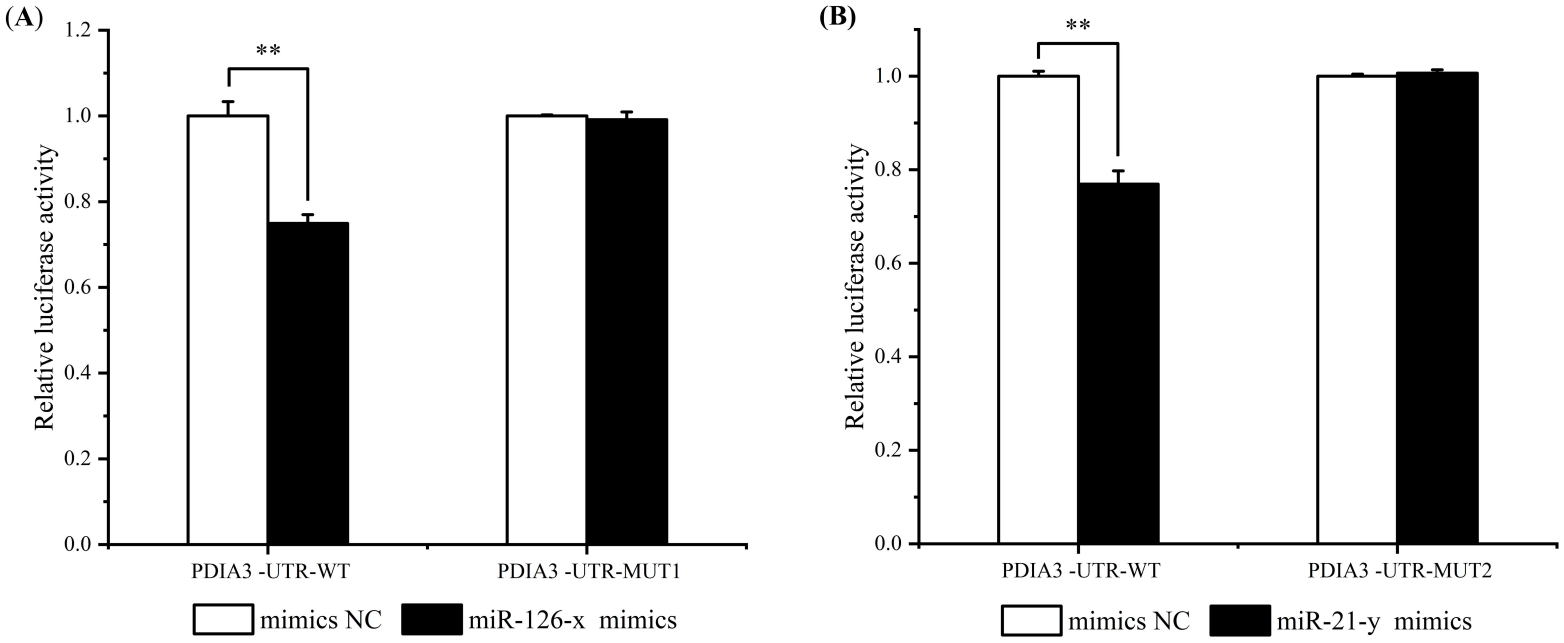
Dual luciferase detection of miR-126-x and miR-21-y regulation of ChPDIA3. **(A)** represents the miR - 126 - x mimics group, showing the luciferase activity of HEK293T cell lines co - transfected with miR - 126 - x mimics and pmirGLO - PDIA3 - 3’ - UTR. **(B)** represents the miR - 21 - y mimics group, indicating the luciferase activity of HEK293T cell lines co - transfected with miR - 21 - y mimics and pmirGLO - PDIA3 - 3’ - UTR. “*” indicates an extremely significant difference between groups (P < 0.01).

The luciferase activities of miR-126-x mimics, miR-21-y mimics and their pmirGLO-PDIA3-3’-UTR co-transfected HEK293T cell lines were assayed by dual luciferase experiments to validate the targeting relationship between miR-126-x, miR-21-y and *ChPDIA3*, respectively. The results showed that both miR-126-x and miR-21-y inhibited *ChPDIA3*. In the miR-126-x group, compared with mimics NC (control group), luciferase activity extremely significantly decreased (*P*<0.01), being 74.93% of the mimics NC (control) group’s level. Mutating the predicted site, the luciferase activity was restored to 99.13% of the control (*P>*0.05) (**Figure 5A**). In the miR-21-y mimics group, dual luciferase activity extremely significantly (*P*<0.01), dropping by 76.94% compared to the control group. Mutating the predicted site restored the luciferase activity to 100.68% of the control (*P>*0.05) (**Figure 5B**).

## 4 Discussion

### 4.1 Bioinformatics analysis of *ChPDIA3*

*PDIA3*, also known as 1,25D3-MARRS or ERp57, is a member of the PDI family, which functions as an endoplasmic reticulum-based redox chaperone protein that interacts with various fibrosis-related proteins. In the endoplasmic reticulum, *PDIA3* acts as a molecular chaperone and redox catalyst to regulate glycoprotein folding (Oliver et al., 1999). In our previous study, *PDIA3* was significantly enriched in the protein processing in endoplasmic reticulum pathway (Hou et al., 2023). In this study, the cDNA sequence of *PDIA3* from the *C. hongkongensis*, was obtained by RACE cloning technology. The homogeneous analysis demonstrated that the nucleic acid sequence and amino acid sequence of this gene are relatively conserved, especially conserved site Thioredoxin_CS (Fritz-Wolf et al., 2011; Yang et al., 2024). It was hypothesized that *ChPDIA3* was identified as a homologous gene of *PDIA3* in the *C. hongkongensis*. Therefore, the mechanism of *ChPDIA3* might be similar to that of other species that have been studied. In addition, in shellfish and crustaceans, the *ChPDIA3* sequence contains the PDI_thioredoxin-like_dom conserved domain, whereas the PDIA3_PDI_b conserved domain is also present in fish and mammals. This difference may indicate some evolution and differentiation in the structure and function of the PDI_thioredoxin-like_dom conserved domain in the *PDIA3* sequences of different species. The PDI_thioredoxin-like_dom conserved domain is usually associated with the function of protein disulfide isomerase (PDI), which plays an important role in the endoplasmic reticulum, helping proteins to fold correctly and form disulfide bonds (Mahmood et al., 2021). However, differences in the conserved domains in the *PDIA3* sequences of different species may reflect differences in their functional requirements or adaptive strategies for this conserved domain during evolution. Differences in the conserved domains in the *PDIA3* sequences of different species may imply that they have diverged in functions such as protein folding and endoplasmic reticulum stress response. These species may have evolved specific *PDIA3* structures and functions during adaptation to different environments and lifestyles (Liu et al., 2022; Trnková et al., 2013). When they are exposed to specific endoplasmic reticulum stress conditions or protein folding requirements, which contribute to the alteration of the *PDIA3* sequence and the evolution of the conserved domains (Huang et al., 2009; Pinto et al., 2013).

Hydrophilicity plays an important role in the immune response (Boquet et al., 1995). It was found that there was a significant hydrophilic complementation between the complementary determining regions (CDR) of anti-neuropeptide substance monoclonal antibody (mAb SP31) and the C-terminal pentapeptide epitope of SP. Peptides that retained the hydrophilicity feature were recognized by mAb SP31 with reduced affinity even though the sequence differed from that of SP, whereas peptides with altered hydrophilicity feature showed a significant decrease in antibody binding affinity or even failed to bind. In addition, the degree of hydrophilic complementarity was closely related to the binding affinity, with peptides with similar ydrophilic profiles having a higher binding affinity to mAb SP31, while peptides with large differences in hydrophilicity showed significantly lower affinity (Hanin et al., 1997). Kinetic studies also suggest that hydrophilic complementarity is important in maintaining the stability of antigen-antibody complexes and may regulate binding affinity by affecting conformational changes and interaction stability after complex formation (Bhat et al., 1994; Van Oss, 1995). hydrophilicity also plays an important role in the immunomodulation of viral infections. The β-convex trigger loop region (QGEESND) of interleukin-1β (IL-1β) is hydrophilic and complementary to specific peptides (e.g., LITVLNI) in the interleukin-1 type I receptor (IL-1R1), and this complementarity influences viral binding to the receptor as well as the subsequent immune response (Heal et al., 1999). In the present study, ChPDIA3 is a hydrophilic protein, and we speculated that ChPDIA3 may play important roles in immune responses through various mechanisms, such as participating in antigen processing and presentation, regulating immune cell activity, influencing cytokine production and secretion, and interacting with other immune-related molecules, thus positively affecting the body’s immune defenses and maintenance of immune homeostasis. However, these roles still need further experimental validation and in-depth studies.

### 4.2 Expression patterns of *ChPDIA3*

Due to its function as a molecular chaperone, *PDIA3* expression rises when cells are stressed. It has been reported that *PDIA3* can trigger Bak-dependent apoptosis by affecting mitochondrial outer membrane permeability (Zhao et al., 2015). Moreover, *PDIA3* has functions such as regulating inflammation and oxidative stress (Wang, 2019) and inhibiting cancer cells (Yang et al., 2024). *PDIA3* also plays an important role in immune activity in aquatic animals. Immunity-related proteins such as *PDIA3* were significantly up-regulated in the intestine of *Cynoglossus semilaevis* in response to *Shewanella algae* infection (Han et al., 2020). The expression level of *ERp57* protein in *O. mykiss* was elevated in peripheral blood leukocytes and RTS11 macrophage-like cell lines in response to stimulation with double-stranded RNA and phytohaemagglutinin, which also suggests a possible conserved function of *ERp57* in the endoplasmic reticulum and activation of immune responses in *O. mykiss* (Sever et al., 2013). This may be due to the respiratory, filter feeding and immunological effects of the gills of the *C. hongkongensis* (Xie et al., 2023; Yue et al., 2024). This makes it possible for *C. hongkongensis* to be frequently exposed to externally supported disease-causing microorganisms such as bacteria and viruses. The gills of the *C. hongkongensis*, as respiratory organs, are the first to come into contact with the outside world, which also makes the gills of the *C. hongkongensis* play an irreplaceable role in its immune defense activities. The expression of *ChPDIA3* was significantly higher in *V. harveyi* and LPS infections relative to controls.

Mollusks typically possess a relatively unsophisticated adaptive immune system, thus relying predominantly on their innate immunity to counteract against foreign pathogens (Chakroun et al., 2021; Tincu and Taylor, 2004; Yang et al., 2021). The innate immune response is characterized by its swift activation, commencing promptly during the initial phase of acute infections(L. Li et al., 2024; Olasard et al., 2024). *V. harveyi*, a Gram-negative bacterium, and LPS, a component of the Gram-negative bacterial cell wall, are both potent activators of the innate immune response (Shi et al., 2024; Yao et al., 2024). They can stimulate pattern recognition receptors (PRR), such as Toll-like receptors (TLRs) (Kawai and Akira, 2010). This recognition mechanism can trigger a rapid inflammatory response, which may include the upregulation of ChPDIA3 as part of the cellular stress response to invading pathogens (Mahmood et al., 2021).In our study, focusing on the *C. hongkongensis*, we had observed the highest expression levels of *ChPDIA3* at 12 hours post-infection with *V. harveyi* and *LPS*. This had suggested that during the incipient stages of infection, *C. hongkongensis* may have bolstered its defense mechanisms by enhancing the proper folding and maturation of proteins critical for immune function, such as antimicrobial peptides (Yoo et al., 2019). Additionally, the early phase of bacterial invasion was likely to have provoked oxidative stress, which in turn, may have activated the unfolded protein response (UPR). Given that *ChPDIA3* had played a role in modulating oxidative stress through its interactions with other oxidoreductases (Chamberlain et al., 2019), its heightened expression during the early infection stages may have served as a cellular protective response to oxidative challenges. However, our sampling regimen, initiated at 12 hours post-infection, precluded a more precise determination of the peak expression timing of *ChPDIA3*. In future related studies, we will consider collecting samples at earlier time points after infection. This will help us more accurately depict the changing trend of the expression level of *ChPDIA3*, so as to evaluate the importance of *ChPDIA3* in the early immune response. Furthermore, we observed a significant downregulation of *ChPDIA3* expression in *C. hongkongensis* that had been treated with Poly(I:C). This phenomenon may have been associated with the immune pathways activated by viral infections, which might not have relied on the function of *ChPDIA3*. For instance, RNA sensing pathways played a primary role in response to viral infections (Kourko et al., 2023; Yue et al., 2023), while *ChPDIA3* may have been more involved in the immune response to bacterial infections. These findings suggested that *ChPDIA3* may have played distinct roles in response to different immune challenges, and its expression regulation could have been closely linked to signaling pathways specific to certain pathogens.

The endoplasmic reticulum protein processing pathway is of crucial significance in the folding, modification, and transport of proteins within cells. *PDIA3*, being a vital member of this pathway, interacts with downstream genes that are indispensable for maintaining the normal physiological functions of cells. In this study, when the *ChPDIA3* was silenced, the expression of the downstream genes of the *ChPDIA3* in the endoplasmic reticulum protein processing pathway was successively down-regulated. We hypothesized that in response to *V. harveyi* infection, *ChPDIA3* might have adjusted the protein processing and folding process by regulating the expression of downstream genes to enhance cellular defenses or adapt to infection-induced stress responses. For example, *CALR* (calreticulin), which was involved in protein folding and quality control, and changes in its expression might have affected the correct folding and stability of intracellular proteins, thereby influencing the cellular immune response and stress resistance (Galluzzi and Kroemer, 2022; Ziffels et al., 2019). *LMAN1* (mannose-binding lectin-associated serine protease 1) and *LMAN2* (mannose-binding lectin-associated serine protease 2), which played a role in innate immunity, and the down-regulation of their expression might have affected the ability of oysters to recognize and clear pathogens (Kwon et al., 2016; Zhang et al., 2023; Zheng et al., 2010). This further emphasized the importance of *ChPDIA3* in the immune defense system of the *C. hongkongensis* and its critical position in the complex regulatory network in response to *V. harveyi* infection.

### 4.3 Regulation of *ChPDIA3* by miR-126-x and miR-21-y

As a unique endothelial cell-associated miRNA, as well as a novel tumour suppressor gene, miR-126 plays a key role in the onset, progression and metastasis of various types of cancers, including hepatocellular carcinoma, colorectal carcinoma, melanoma and lung cancer. For example, cellular processing of MPM-derived spheroids by exosomal delivery of miR-126 leads to massive cell death and prevents tumour growth *in vivo* (Monaco et al., 2022). Overexpression of miR-126-3p in ovarian cancer cells inhibited cell proliferation and invasion as well as phosphorylation of *AKT* and *ERK1/2* (Xiang and Cheng, 2018). The miR-21 family includes members such as miR-21-5p and miR-21-3p. These miRNAs play important regulatory roles in cell growth, differentiation, apoptosis and other processes, and play an important role in the treatment of cancer. The exosome miR-21-5p in hepatocellular carcinoma cells can affect hepatocellular carcinoma cell development and patient prognosis by regulating *SP1/XBP1* and promoting M2 polarization in TAMs (Hu et al., 2024). miR-21 is elevated in canine mammary tumors and positively correlates with gene expression of *IL-6* and *TNF-α* and also with the proliferation index (Ki67 index) of tumour cells (Abbate et al., 2023). In this study, the binding sites of miR-126-x, miR-21-y and *ChPDIA3* were detected using dual luciferase experiments, respectively. The results showed that both miR-126-x and miR-21-y inhibited the 3’-UTR region of *ChPDIA3*. This suggested that they might be involved in the regulation of *ChPDIA3* gene expression. And the expression of *ChPDIA3* may be related to the response of *C. hongkongensis* to *V. harveyi* infection. When faced with *V. harveyi* infection, the organism may affect the expression of *ChPDIA3* by regulating the expression levels of miR-126-x and miR-21-y, thereby modulating the immune response or other relevant physiological processes of the organism in response to the infection. This regulatory mechanism helps the *C. hongkongensis* to better resist *V. harveyi* infection and maintain the health and homeostasis of the organism.

### 4.4 Potential applications in aquaculture

Our findings on the role of *ChPDIA3* in *C. hongkongensis* and its regulation by miR-126-x and miR-21-y offer potential applications for the aquaculture industry, particularly in enhancing disease resistance in oysters. One promising application is through selective breeding programs. By identifying and selecting oysters with higher levels of *ChPDIA3* expression or those with genetic variants that enhance the gene’s function, breeders can develop lines with greater resistance to bacterial infections, such as those caused by *V. harveyi*. This approach will enable the breeding of oyster strains that are more resilient to disease outbreaks and reduce reliance on antibiotics, thereby promoting more sustainable and environmentally friendly aquaculture practices (Chamberlain et al., 2019; Wang et al., 2023). Additionally, the manipulation of miRNA expression levels, such as miR-126-x and miR-21-y, can be explored as a genetic engineering strategy. By regulating these miRNA levels, it is possible to increase the expression of *ChPDIA3* and other immune-related genes, thereby enhancing the overall immune response in oysters (Chu et al., 2021; Pan, 2024; Qin et al., 2024). This could be particularly useful in hatcheries and nurseries, where oysters are most susceptible to infections. In conclusion, our study may contribute to the selection of disease-resistant oyster strains and disease control. However, the results of the current study are still some distance from direct application in production and require further in-depth research.

### 4.5 Limitations of this study

While our study provides new insights into the role of *ChPDIA3* and its regulation by miR-126-x and miR-21-y in the immune response of *C. hongkongensis* against *V. harveyi* infection, several limitations should be acknowledged. First, the experimental design primarily focused on laboratory conditions, which may not fully replicate the complex environmental factors encountered in natural aquaculture settings. Factors such as water temperature, salinity, and the presence of other pathogens can significantly influence the expression and function of immune-related genes. Second, the study utilized a limited number of time points for sampling, which may not capture the complete dynamics of the immune response over the entire course of infection. Future studies would likely necessitate more frequent sampling to better understand the temporal changes in gene expression and the potential involvement of other immune pathways. Additionally, while the dual luciferase experiments provided evidence of miRNA regulation, the study did not explore the downstream effects of *ChPDIA3* on other components of the immune system in detail, such as signaling pathways. Further research is needed to elucidate the broader network of interactions and pathways involved in the immune response mediated by *ChPDIA3*.

## 5 Conclusion

The *PDIA3* gene has been identified to play an important role in the infection of *C. hongkongensis* infected under *V. harveyi*. In this paper, the *PDIA3* sequence of the *C. hongkongensis* was cloned. The homogeneous analysis demonstrated that the nucleic acid sequence and amino acid sequence of this gene are relatively conserved. It is proved that *ChPDIA3* has a similar mechanism to other homologous genes. *ChPDIA3* was most highly expressed in gill tissues of the *C. hongkongensis* as detected by qPCR, and its expression was higher than that of Poly (I:C) in the presence of *V. harveyi* and LPS infections. This may indicate that *ChPDIA3* is primarily involved in the immune response against bacterial infections in the *C. hongkongensis*. Dual luciferase reported that both miR-126-x and miR-21-y inhibited the expression of *ChPDIA3*. In practical application, the expression level or gene sequence polymorphism of *ChPDIA3* gene can be used as an important molecular marker for disease resistance selection, and it is expected to breed oyster varieties with stronger disease resistance by selecting individuals with specific *ChPDIA3* expression characteristics for breeding.

## Data Availability

The original contributions presented in the study are publicly available. This data can be found here: GenBank: PP530092.
